# 1251. 5-year outcomes of bictegravir/emtricitabine/tenofovir alafenamide (B/F/TAF) as initial treatment of HIV-1 in adults with high baseline HIV-1 RNA and/or low CD4 count in two Phase 3 randomized clinical trials

**DOI:** 10.1093/ofid/ofac492.1082

**Published:** 2022-12-15

**Authors:** Moti Ramgopal, Anson Wurapa, Axel Baumgarten, Mezgebe Berhe, Anton Pozniak, Chloe Orkin, Juan Manuel Tiraboschi, Debbie P Hagins, Hailin Huang, Kristin Andreatta, Nathan Unger, Jason Hindman, Hal Martin, Jared Baeten, Olayemi Osiyemi

**Affiliations:** Midway Specialty Care Center, Fort Pierce, Florida; Infectious Disease Specialists of Atlanta, Decatur, Georgia; Center for Infectious Diseases (zidp), Berlin, Berlin, Germany; North Texas Infectious Diseases Consultants, Dallas, Texas; Chelsea and Westminster Hospital, London, England, United Kingdom; Queen Mary University of London, London, England, United Kingdom; Bellvitge University Hospital, Barcelona, Catalonia, Spain; Georgia Department of Public Health, Coastal Health District, Chatham CARE Center, Savannah, GA, USA, Savannah, Georgia; Gilead Sciences, Inc., Foster City, California; Gilead Sciences, Foster City, California; Gilead Sciences, Foster City, California; Gilead Sciences, Foster City, California; Gilead Sciences, Foster City, California; Gilead Sciences, Foster City, California; Triple O Research Institute, West Palm Beach, Florida

## Abstract

**Background:**

People with HIV (PWH) who are initiated on guidelines-recommended first-line INSTI-based antiretroviral therapy routinely achieve rapid virologic suppression; however, those with a high baseline (BL) HIV-1 RNA and/or low CD4 count may be more challenging to manage in the short- and long-term. To further characterize long-term outcomes over 5 years in select subgroups, we analyzed results from two studies examining B/F/TAF as initial treatment stratified by BL HIV-1 RNA and/or CD4 count.

**Methods:**

Adults with HIV were randomized to receive blinded initial treatment with B/F/TAF versus dolutegravir [DTG]/abacavir/lamivudine (Study 1489) or DTG+F/TAF (1490) for 144 weeks (W) of blinded treatment followed by an optional switch to open-label B/F/TAF for 96W. We present virologic response (HIV-1 RNA < 50 c/mL, missing=excluded and missing=failure) and study drug-related adverse events (DRAE) from a pooled analysis of participants originally randomized to B/F/TAF who had BL HIV-1 RNA 100,00-400,000 copies(c)/mL, HIV-1 RNA >400,000 c/mL and/or CD4 count < 200 cells/µL through W240.

**Results:**

634 adults (median age 32 years, 89% men, 33% Black/African descent, 24% Hispanic/LatinX) originally randomized to B/F/TAF were included for analysis. At BL, 80 participants had a BL CD4 count < 200 cells/µL and 119 participants had HIV-1 RNA >100,000 c/mL, of whom, 20 had HIV-1 RNA >400,000 c/mL. At W240, virologic suppression was high for the low CD4 count and/or high HIV-1 RNA subgroups (Table). No participant in the final resistance analysis developed virologic resistance to any component of B/F/TAF. Across the subgroups, the most common DRAEs were nausea, headache and diarrhea and there were no serious DRAEs. There was only one discontinuation due to a DRAE in the low CD4 count subgroup, and none in the high HIV-1 RNA subgroup.

**Figure:**
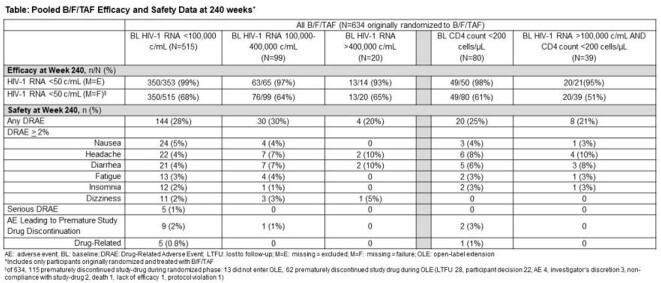

**Conclusion:**

Initial treatment with B/F/TAF was safe and efficacious over 5 years of follow-up in people with a high BL HIV-1 RNA and/or low CD4 count. These outcomes provide additional evidence that B/F/TAF is an effective and durable regimen for a broad range of PWH, including those with advanced disease.

**Disclosures:**

**Moti Ramgopal, MD, FACP, FIDSA**, Gilead Sciences: Advisor/Consultant|Gilead Sciences: Speakers Bureau|Janssen: Advisor/Consultant|Janssen: Speakers Bureau|Merck: Advisor/Consultant|Merck: Speakers Bureau|ViiV: Advisor/Consultant|ViiV: Speakers Bureau **Axel Baumgarten, MD**, AbbVie: Honoraria|Gilead Sciences: Honoraria|Janssen: Honoraria|MSD: Honoraria|ViiV: Honoraria **Anton Pozniak, MD, FRCP**, Gilead: Grant/Research Support|Gilead: Honoraria|Janssen: Grant/Research Support|Janssen: Honoraria|Merck: Honoraria|theratec: Honoraria|ViiV: Grant/Research Support|ViiV: Honoraria **Chloe Orkin, MBChB, FRCP, MD**, Gilead Sciences: Honoraria|GSK: Honoraria|Janssen: Honoraria|MSD: Honoraria **Juan Manuel Tiraboschi, PhD**, Gilead Sciences: Advisor/Consultant|Gilead Sciences: Grant/Research Support|Janssen: Advisor/Consultant|Janssen: Grant/Research Support|MSD: Advisor/Consultant|MSD: Grant/Research Support|ViiV Healthcare: Advisor/Consultant|ViiV Healthcare: Grant/Research Support **Debbie P. Hagins, MD, FAPCR, AAHIVS**, Gilead Sciences: Advisor/Consultant|Gilead Sciences: Grant/Research Support|Gilead Sciences: Speakers Bureau|Janssen: Grant/Research Support|Merck: Advisor/Consultant|Merck: Grant/Research Support|ViiV: Advisor/Consultant|ViiV: Grant/Research Support **Hailin Huang, PhD**, Gilead Sciences, Inc.: Employer|Gilead Sciences, Inc.: Stocks/Bonds **Kristin Andreatta, MSc**, Gilead Sciences, Inc: Employee of Gilead Sciences|Gilead Sciences, Inc: Stocks/Bonds **Nathan Unger, PharmD, AAHIVP**, Gilead Sciences: Employee|Gilead Sciences: Stocks/Bonds **Jason Hindman, PharmD, MBA**, Gilead Sciences: Employee|Gilead Sciences: Stocks/Bonds **Hal Martin, MD**, Gilead Sciences: employee|Gilead Sciences: Stocks/Bonds **Jared Baeten, MD, PhD**, Gilead Sciences: Employee|Gilead Sciences: Stocks/Bonds **Olayemi Osiyemi, MD**, Gilead: Advisor/Consultant|gsk: Advisor/Consultant|viiv: Advisor/Consultant.

